# Latent profile analysis and analytical model construction based on heterogeneity of intrinsic capacity in Chinese older adults

**DOI:** 10.3389/fnagi.2026.1858286

**Published:** 2026-07-28

**Authors:** Dewei Xu, Yanyu Jiang, Hao Liu, Yanlin Leng, Jason Xiong, Hui Wang, Zhaoxia Wang, Junfeng Wang, Yong Tang

**Affiliations:** 1 College of Computer Science, Sichuan University, Chengdu, China; 2 School of Computer Science and Engineering, University of Electronic Science and Technology of China, Chengdu, China; 3 School of Information Technology, Zhejiang Financial College, Hangzhou, China; 4 Department of Computer Information Systems, Walker College of Business, Appalachian State University, Boone, NC, United States; 5 Department of Neurology, Peking University First Hospital, Beijing, China; 6 Shuanghua Digital Health and Intelligent Medicine Research Institute, Chengdu, China; 7 Institute of Intelligent Chinese Medicine, Chongqing University of Chinese Medicine, Chongqing, China; 8 International Research Center for Complexity Sciences, Hangzhou International Innovation Institute, Beihang University, Hangzhou, China

**Keywords:** community health assessment, intrinsic capacity, latent profile analysis, psychological health, XGBoost

## Abstract

**Background:**

Intrinsic capacity (IC) is a critical multidimensional indicator of healthy aging; however, current research on IC primarily emphasizes its longitudinal trajectories, with limited recognition of the heterogeneity within older adults and a notable lack of targeted assessment instruments. This study aimed to identify distinct IC profiles among Chinese older adults and explored an interpretable, web-based analytical model to enable rapid profiles classification.

**Methods:**

A cross-sectional survey was conducted across 24 provinces in China, enrolling 4,508 older adults from community and institutional care settings via stratified sampling. IC scores were assessed using the MNA-SF, SPPB, and GDS-15. Latent profile analysis (LPA) was employed to delineate IC subgroups. Feature selection integrated univariate regression, random forest, and LASSO regression. An XGBoost algorithm was subsequently trained (70%) to predict profile membership and validated on an independent test set (30%). Model interpretability was enhanced using SHAP and XGBoost gain values. Furthermore, an interactive web-based platform was developed using Python (version 3.9), JavaScript (version 7), and MySQL (version 8.0).

**Results:**

Based on their five-domain IC scores, two latent profiles were identified: “Overall Low IC” and “Overall High IC” (Entropy = 0.846, *P* < 0.05). Key predictors selected included self-rated health (SRH) (*OR* = 1.26, 95% *CI*: 1.17–1.36), age (*OR* = 0.87, 95% *CI*: 0.80–0.95), presence of multiple chronic diseases (*OR* = 1.15, 95% *CI*: 1.05–1.25), chest tightness and pain (*OR* = 1.28, 95% *CI*: 1.02–1.62), and mobility issues (*OR* = 2.54, 95% *CI*: 2.08–3.11). The XGBoost model demonstrated robust discriminative performance for the “Overall High IC” profile, achieving an AUC of 0.910 (95% *CI*: 0.898–0.921) and accuracy of 0.874 (95% *CI*: 0.862–0.885) in the training set, and an AUC of 0.853 (95% *CI*: 0.828–0.875) with accuracy of 0.819 (95% *CI*: 0.800–0.838) in the test set. Decision curve analysis (DCA) confirmed a clinical net benefit within a threshold probability range of 4%−97%. The deployed web-based platform generates stratified predictions within 3 s and demonstrates clinical interpretability at the individual level.

**Conclusion:**

This study delineates two IC profiles among Chinese older adults. The XGBoost-based model was explored for rapid IC profile classification. These findings highlight heterogeneity in IC and support the feasibility of a simplified digital approach, with external validation warranted.

## 1 Introduction

The global proportion of older adults (aged over 60) has been steadily rising in recent years (WHO, 2020). Notably, China is experiencing rapid population aging while hosting the world's largest population, comprising roughly one-fifth of the elderly population worldwide (WHO, 2020; Chen et al., 2022). Between 2010 and 2024, the proportion of older adults in China increased from 13.26% to 22% (Communiqué of the National Bureau of Statistics of People's Republic of China on Major Figures of the 2010 Population Census[1] (No. 1), 2011; Statistical Communiqué of the People's Republic of China on the 2024 National Economic and Social Development, 2025). This demographic transition presents significant economic challenges, underscoring the imperative to promote healthy aging (Chen et al., 2022).

Healthy aging extends beyond the mere absence of disease or the maintenance of physical health (WHO, 2022), it is a holistic concept that emphasizes not only longevity but also well-being and quality of life in later years. The World Health Organization (WHO) has proposed an innovative model designed to reshape geriatric care, shifting the focus from a disease-centered approach to a function-centered paradigm. Central to this model is the concept of intrinsic capacity (IC), the composite of all physical and mental capacities that an individual can draw upon at any point in life (Cesari et al., 2026). IC encompasses five key domains: cognition (the ability to think, learn, remember, and make decisions.), vitality (the body's energy reserve and nutritional status.), locomotion (the ability to move, walk, and maintain balance.), psychological (emotional well-being and the absence of depressive symptoms.), and sensory (the function of vision and hearing.) (Xuan et al., 2026; World Health Organization, 2017). To facilitate the implementation of this model, the WHO published the Integrated Care for Older People (ICOPE) framework, which offers standardized guidance for the comprehensive assessment of IC. In 2021, the WHO formally recognized age-related decline in intrinsic capacity under the ICD-11 code MG2A (Peng et al., 2026). The WHO (2022) further emphasized that functional ability is shaped by intrinsic capacity, environmental factors, and their dynamic interactions. In summary, IC is regarded not only as the foundation of functional ability but also as a core determinant in evaluating healthy aging (Chhetri et al., 2022).

Most existing studies measure IC level based on the five domains proposed by Cesari et al. (2018) (vitality capacity, sensory capacity, locomotor capacity, cognitive capacity, and psychological capacity) and further classify IC or explore its associations with various factors (e.g., physical activity, aging-related biomarkers) (Zhang et al., 2025; Lu et al., 2023; Zhang et al., 2024). However, these studies mainly focus on the overall IC score, overlooking the variability across individual domain scores. Given the multidimensional nature of health in older adults, relying solely on the overall IC score constrains the capacity to provide detailed and targeted intervention guidance. Thus, applying latent profile analysis (LPA) or latent class analysis (LCA) to identify potential latent classes and their associated factors is important. Although few studies have applied LCA, the participants were typically drawn from specific populations (e.g., rural older adults, hospitalized older adults) in certain regions of China (Yu et al., 2022; Wang et al., 2025). Moreover, LCA focuses on discrete classes and does not capture continuous patterns, limiting its ability to fully characterize the heterogeneity of IC. Despite these prior efforts, the use of LPA to identify latent IC profiles and to compare the associated factors with those derived from LCA in broader populations remains insufficiently explored.

In addition, researchers have primarily relied on the China Health and Retirement Longitudinal Study (CHARLS) dataset in studies of the older population in China. Although it includes 17,708 participants, only about 2,000–5,000 older people are typically included in IC analyses (Zhao et al., 2014; Zhang et al., 2025; Yue et al., 2025). By the end of 2023, the older population in China had reached approximately 297 million, and the CHARLS dataset represents only a tiny proportion of the older population. Moreover, the most recent wave of the CHARLS dataset was collected in 2020, and may not adequately capture recent societal changes. Therefore, there is an urgent need to increase the participation of older individuals and update the data for the IC analysis.

XGBoost is widely used machine learning algorithm for binary classification (Silva et al., 2022; Smiley et al., 2025) and has recently been applied in IC-related studies (Sun et al., 2025; Cruz-Montecinos et al., 2025). Compared with linear regression models or decision trees, its boosting ensemble captures complex non-linear relationships between features and the outcome, improving model generalizability and stability. It also supports parallel computation for faster training and provides built-in feature importance measures, which facilitate model interpretability (Chen and Guestrin, 2016). Its compact model size and fast inference further support its suitability for deployment in web-based applications. Therefore, XGBoost shows potential for application in the development of an online IC assessment platform.

Accordingly, first, this study includes 4,508 older adults from 24 provinces in China and aims to identify the latent profiles of IC, as well as compare their associated factors with those reported in previous studies among broader populations. Second, an interactive online IC assessment platform is explored based on the XGBoost model, which may provide preliminary methodological evidence for the potential of digital classification of IC latent profiles.

## 2 Materials and methods

### 2.1 Participants and study design

This study was conducted between December 2023 and December 2024 across 24 provinces in China, encompassing participants from both residential communities and nursing facilities. A community-based, cross-sectional, observational survey design was applied to systematically capture data from older adults across multiple geographic regions. To ensure broad geographic and demographic representation, a stratified random sampling method was employed, with local health agencies collaborating to enhance community engagement and maximize participation rates.

The inclusion criteria were: (1) age ≥60 years; (2) provision of written informed consent, either personally or through a legal guardian; and (3) ability to complete a standardized intrinsic capacity (IC) assessment. Exclusion criteria included: (1) presence of acute medical illness or end-of-life status; and (2) inability to complete the assessment due to severe cognitive or physical limitations.

Following the study of Ma et al. (2017), we utilized statistical sampling techniques, including clustering, stratification, and random selection. This multi-stage sampling approach was executed sequentially based on geographical distribution, stratifying first by northern and southern regions, and subsequently by urban and rural areas. Specifically, in the first stage, representative cities were selected. From the northern region, 42 representative cities were selected, including Beijing (North China), Xi'an (Northwest China), Harbin (Northeast China), Changchun (Northeast China), Hohhot (North China), Yinchuan (Northwest China), Xining (Northwest China), Lanzhou (Northwest China), Urumqi (Northwest China), Shijiazhuang (North China), Taiyuan (North China), and Shenyang (Northeast China). From the southern region, 55 representative cities were selected, including Chengdu (Southwest China), Chongqing (Southwest China), Changsha (Central China), Shanghai (East China), Nanjing (East China), Hangzhou (East China), Hefei (East China), Nanchang (East China), Jinan (East China), Zhengzhou (Central China), Wuhan (Central China), Guangzhou (South China), Nanning (South China), Kunming (Southwest China), and Guiyang (Southwest China). In total, these 97 cities spanned 24 provincial-level administrative regions, covering the seven major geographical divisions of mainland China: North China (e.g., Beijing, Tianjin, Shijiazhuang), Northeast China (e.g., Harbin, Changchun, Shenyang), East China (e.g., Shanghai, Nanjing, Hangzhou), Central China (e.g., Changsha, Zhengzhou), South China (e.g., Nanning, Liuzhou), Southwest China (e.g., Chengdu, Chongqing, Kunming), and Northwest China (e.g., Xi'an, Lanzhou, Urumqi). In the second stage, older adults residing in different urban and rural areas within each selected city were recruited and stratified by gender, with the sex ratio aligned with the demographic profile from the Seventh National Population Census (2020).

Among the total 4,511 recruited participants, 2,346 were male and 2,165 were female (sex ratio: 1.08, approximating the national census ratio of 1.05). Three participants were excluded due to missing disability data, resulting in a final analytical cohort of 4,508 older adults aged 60 years and older, with a valid ratio of 99.9%. All participants provided written informed consent prior to enrollment.

**Figure 1:**
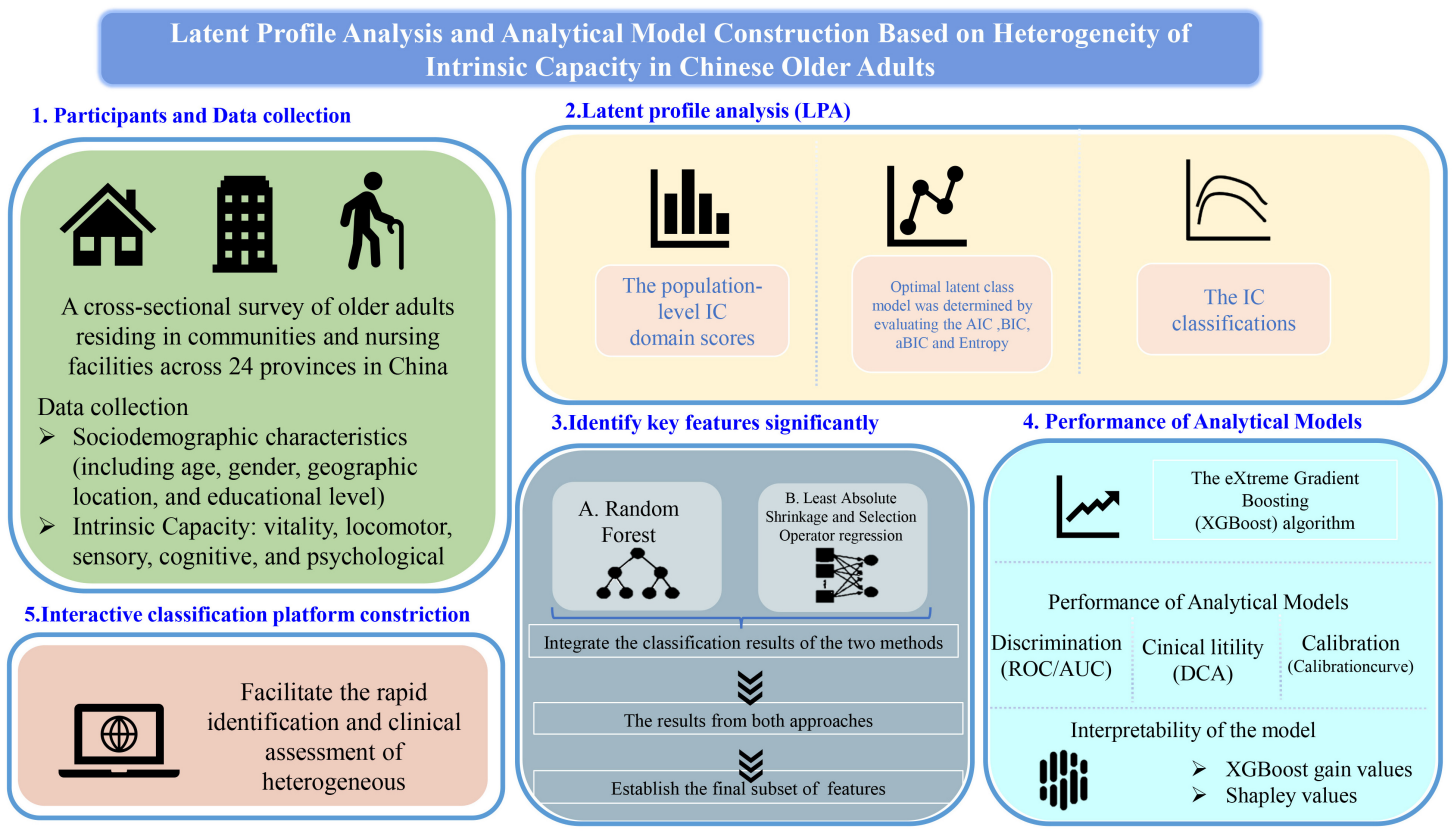
Research workflow overview. Infographic outlining the research workflow for latent profile analysis and predictive model construction of intrinsic capacity in Chinese older adults.

The study protocol was approved by the Ethics Committee of Peking University First Hospital (Approval No.2023-Y-564-001). All procedures were conducted in accordance with relevant regulations and the ethical principles outlined in the Declaration of Helsinki. The study flowchart is shown in Figure 1.

### 2.2 Assessment of intrinsic capacity

IC was evaluated across five domains defined by Cesari et al. (2018), including vitality, sensory, locomotor, cognitive, and psychological capacity. To ensure methodological consistency and comparability across study participants, standardized scales were utilized.

Vitality was quantified using the Mini-Nutritional Assessment-Short Form (MNA-SF) to screen for nutritional risk (score range: 0–14), with lower scores indicating poorer nutritional status. Sensory function encompassed visual and auditory assessments. Visual acuity was measured using a simplified E-chart protocol (score range: 0–6), and hearing was evaluated through pure-tone audiometric screening at frequencies of 500–4,000 Hz. The sensory function score ranges from 0 to 10, calculated as the sum of vision scores (each eye scored from 0 to 3) and hearing scores (each ear scored from 0 to 2) (Leng et al., 2025). The detailed evaluation scheme can be found in Supplementary Material 1. Locomotion was quantified using the Short Physical Performance Battery (SPPB) (Garcia et al., 2024), which assesses gait speed, repeated chair stands, and balance, with total scores ranging from 0 to 12 and higher scores denoting superior motor function. Cognition was assessed using the Mini-Mental State Examination (MMSE) (Tuena et al., 2023), a well-established screening tool for cognitive impairment. Psychological capacity was evaluated via the 15-item Geriatric Depression Scale (GDS-15) (Sheikh and Yesavage, 1986; Mui, 1996; Wancata et al., 2006; Boey, 2000), with higher scores indicating greater depressive symptoms.

### 2.3 Latent profile analysis (LPA)

To identify unobserved subgroups and characterize heterogeneity in IC across individuals, LPA was initially performed using Mplus software (version 8.3) to classify IC levels based on five domains. The optimal latent class model was determined by evaluating the Akaike information criterion (AIC), Bayesian information criterion (BIC), sample-size-adjusted BIC (aBIC), Lo–Mendell–Rubin likelihood ratio test (LMRT), bootstrap likelihood ratio test (BLRT), and entropy. Among these metrics, the AIC, BIC, and aBIC were utilized to evaluate model performance, with lower values indicating a more optimal balance between goodness-of-fit and model complexity. For both LMRT and BLRT, *P* < 0.05 indicated that a *k*-class model provides a significantly better fit than a (*k* – 1)-class model. Entropy, ranging from 0 to 1, measured the classification accuracy, with higher values reflecting greater precision. Conventionally, an entropy value of at least 0.80 indicates robust classification (Sinha et al., 2021). Given that LPA directly models the original distributions of each indicator and does not require cross-indicator comparability, raw scores of the intrinsic capacity domains were retained in the analysis to facilitate the clinical interpretation of the results (Magidson and Vermunt, 2002; Masyn, 2013). Additionally, as a sensitivity analysis, this study also conducted LPA on the standardized data based on the five dimensions of IC.

### 2.4 Model development based on LPA classification

#### 2.4.1 Feature selection

Based on latent profile analysis (LPA), the IC classifications were first obtained, providing a foundation for constructing the model using machine learning. To facilitate robust modeling, feature selection was conducted on the 27 variables derived from the questionnaire survey using both random forest and least absolute shrinkage and selection operator (LASSO) regression. Categorical variables were label-encoded into integer values, whereas continuous variables were retained in their original form. A consistent encoding strategy was applied throughout all subsequent analyses. Participants with missing values were excluded during the inclusion process; therefore, no missing data imputation was required for any variables.

Within the random forest framework, features were ranked by the mean decrease in Gini impurity during node splitting, thereby quantifying their contributions. Concurrently, LASSO regression applied an L1 penalty to shrink the coefficients of less informative features exactly to zero, thereby isolating the most robust features. Finally, by integrating the results of both frameworks, features demonstrating high value across both the random forest and LASSO models were retained as the definitive feature subset to construct the model for IC classification.

#### 2.4.2 Analytical model and interactive platform construction

The dataset was randomly partitioned into a training set (70%) and a test set (30%). The training set was utilized for feature selection and model development, while the independent test set was reserved to evaluate the performance of the final model. Subsequently, an extreme gradient boosting (XGBoost) algorithm was employed to construct an IC classification model using the selected feature subset as input and underwent hyperparameter tuning via a grid search with five-fold cross-validation on the training set. Accuracy was retained as the evaluation metric for the grid search. The hyperparameter combination achieving the highest cross-validation performance was then employed to train the final model, which was evaluated on the independent test set. Then, for facilitating practical application, the XGBoost analytical model was deployed as an interactive tool. User-input characteristics are automatically processed by the system to determine the IC status and the associated probability.

### 2.5 Model performance evaluation

The performance of the IC classification model was evaluated across four domains (discrimination, clinical utility, calibration, and generalization) using 1,000 bootstrap resamples. First, discrimination was evaluated by constructing receiver operating characteristic (ROC) curves and calculating the area under the curve (AUC), sensitivity, specificity, and accuracy. Subsequently, decision curve analysis (DCA) was performed to assess the clinical utility of the model. This analysis calculated the net benefit across threshold probabilities from 0 to 1, quantifying the trade-off between the clinical benefits of true positives and the harms of false positives. Furthermore, to evaluate the reliability of probabilities, calibration curves were generated, and overall model calibration was quantified using the Brier score. Finally, to assess the generalizability of the model, all aforementioned metrics were calculated and compared between the training and independent test sets.

### 2.6 Interpretability of the model

XGBoost gain values and Shapley Additive exPlanations (SHAP) values were used to quantify the priority of key features and to interpret feature contributions in the XGBoost model, respectively. The feature importance function in XGBoost ranks features by gain values in descending order by default, providing a global feature importance ranking. SHAP values were used to evaluate the marginal contribution of each feature by computing Shapley values, thereby assessing the influence of individual features. A beeswarm summary plot based on mean absolute SHAP values was generated to visualize the direction and magnitude of feature effects across all samples, while a SHAP force plot was generated for an individual subject to explain the classification outcome at the individual level. The contributions of the primary variables driving IC classification were visualized through SHAP, effectively enhancing the interpretability and clinical credibility of the model.

### 2.7 Statistical analysis

Statistical analyses were performed using IBM SPSS Statistics (version 26.0) and R (version 4.3.3). An interactive website was developed within the Visual Studio Code cross-platform environment, utilizing Python (version 3.9), JavaScript (version 7), and MySQL (version 8.0).

Descriptive statistics were performed to analyze the initial data. Normally distributed continuous variables were expressed as mean ± standard deviation (SD), whereas non-normally distributed variables were reported as median (interquartile range, IQR). Categorical variables were expressed as frequencies (percentages). Comparisons of variables distribution between IC categories were performed using the independent-samples *t*-test for normally distributed continuous variables, the Mann–Whitney *U*-test for non-normally distributed variables, and the chi-square test or other non-parametric tests for categorical variables. All tests were two-sided, and *P* < 0.05 was considered statistically significant.

## 3 Result

### 3.1 Characteristics of two IC classification identified by LPA

**Figure 2:**
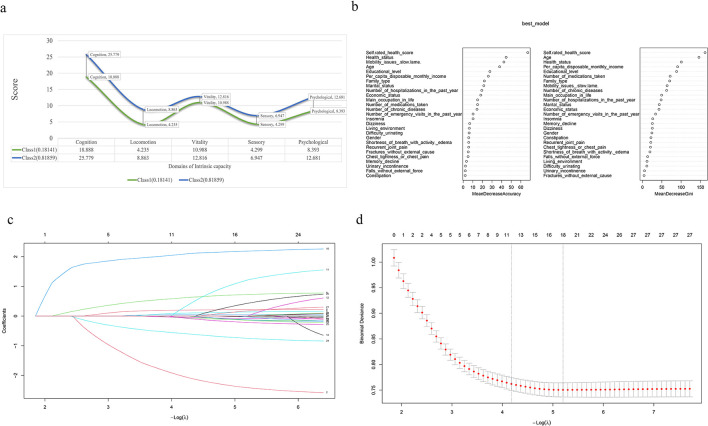
Latent profile analysis and feature selection. **(a)** Line chart comparing domain scores of IC (cognition, locomotion, vitality, sensory, and psychological) between the two latent classes, with a summary table of specific scores shown beneath. **(b)** Variable importance bar plots from the random forest model, with predictors ranked by mean decrease accuracy and mean decrease Gini. **(c)** LASSO regression coefficient paths plotted against the negative log of lambda (λ), with each line representing an individual variable. **(d)** Cross-validation error curve for LASSO regression with standard deviation error bars as a function of λ.

Three latent profile models (Models 1–3), corresponding to one, two, and three latent IC classes, respectively, were constructed using the five IC domains. From Models 1 to Model 3, the AIC, BIC, and aBIC gradually decreased, indicating improved fit with increasing numbers of latent IC classes (Figure 2a and Table 1). Additionally, as a sensitivity analysis, the models on the standardized data based on the five dimensions of IC, which also support the 2-class (Supplementary Figure 1 and Supplementary Table 1). Moreover, both the LMR-LRT and BLRT indicated statistical significance (*P* < 0.05) for Models 1 and 2, whereas the LMR-LRT was not statistically significant for Model 3 (*P* = 0.061), suggesting that a maximum of two classes is appropriate. The Entropy value for Model 2 (0.846) exceeded 0.8, suggesting robust classification. Consequently, the study stratified participants into two latent IC classes: Class 1 (Overall Low IC) and Class 2 (Overall High IC). The current LPA result reflects overall level differences rather than a categorical structure, pointing toward a continuous dimension of IC variation in this population. Meanwhile, in this study, only 5.73% of participants fell in the posterior probability range of 0.70–0.85, indicating a “Classification Uncertainty” zone. The identified profiles were characterized by consistently higher or lower scores across all IC domains. Nevertheless, the LPA-derived profiles were not equivalent to classifications based on mean or median total IC score cut-offs (mean cutoff = 63.27; median cutoff = 66.0) in this study. Specifically, a comparison between the LPA-derived classification and a simple mean-split of the total IC score yielded only moderate agreement (κ = 0.47), with 23.6% of participants classified as “Overall High IC” by LPA despite falling below the total-score cut-off (Supplementary Table 2). In addition, posterior probabilities were estimated for class membership in the LPA, which are not available in mean-based or median-based split approaches. Among them, 5.73% of participants had posterior probabilities between 0.70 and 0.85, indicating a zone of classification uncertainty, as detailed in Supplementary Table 3.

**Table 1:** LPA classification of intrinsic capacity.

Class	AIC	BIC	aBIC	Entropy	LMRT	BLRT	Probability of class
1	148,290.2	148,357.3	148,325.5				
2	143,483.4	143,590.7	143,539.8	0.846	0	0	0.18141/0.81859
3	141,689.4	141,836.9	141,766.9	0.899	0.0613	0	

### 3.2 Participants baseline characteristics

The baseline characteristics of the study population are presented in Table 2. A total of 4,508 participants were included, with 915 (20.3%) classified as “Overall Low IC” and 3,593 (80.7%) as “Overall High IC”. The mean age of participants in “Overall Low IC” and “Overall High IC” was 74.82 ± 7.67 years and 70.57 ± 6.45 years, respectively, with corresponding mean self-rated health scores (SRH) of 4.98 ± 1.65 and 6.91 ± 1.87. Male participants accounted for 42.8% of “Overall Low IC” and 46.8% of “Overall High IC”. Among the participants, 51.3% were community-dwelling and exhibited a low comorbidity burden (median Charlson score: 0; ≥3 chronic conditions: 19.4%), suggesting an underrepresentation of the most functionally compromised older adults, as summarized in Table 2.

**Table 2:** Demographic information of the participants.

Variable	Intrinsic ability category	
	Class 1 (overall low IC)	Class 2 (overall high IC)
*N*	915 (20.30%)	3,593 (80.70%)
Gender = man (%)	392 (42.8)	1,682 (46.8)
Main_occupation_in_life (%)		
Work mainly involving mental labor	137 (15.0)	823 (22.9)
Work mainly involving light physical labor	454 (49.6)	1,952 (54.3)
Work mainly involving heavy physical labor	289 (31.6)	771 (21.5)
Other	35 (3.8)	47 (1.3)
Per_capita_disposable_monthly_income (%)		
< 500 Yuan	176 (19.2)	352 (9.8)
500–999 Yuan	123 (13.4)	359 (10.0)
1,000–1,999 Yuan	188 (20.5)	533 (14.8)
2,000–2,999 Yuan	202 (22.1)	691 (19.2)
3,000–5,999 Yuan	177 (19.3)	1,221 (34.0)
6,000–9,999 Yuan	41 (4.5)	392 (10.9)
≥10,000 Yuan	8 (0.9)	45 (1.3)
Economic_status (%)		
Good	260 (28.4)	422 (11.7)
Middle	511 (55.8)	2,278 (63.4)
Poor	144 (15.7)	893 (24.9)
Marital_status (%)		
Unmarried	12 (1.3)	20 (0.6)
Married and living together	599 (65.5)	2,898 (80.7)
Married and separated	28 (3.1)	129 (3.6)
Divorced	8 (0.9)	49 (1.4)
Widowed	257 (28.1)	489 (13.6)
Others	11 (1.2)	8 (0.2)
Family_type (%)		
Living alone	137 (15.0)	318 (8.9)
The couple	310 (33.9)	1,928 (53.7)
Two generations	225 (24.6)	571 (15.9)
Three generations	219 (23.9)	719 (20.0)
Generations apart	4 (0.4)	30 (0.8)
Other	20 (2.2)	27 (0.8)
Educational_level (%)		
Illiterate	210 (23.0)	314 (8.7)
Primary school	325 (35.5)	816 (22.7)
Junior high school	200 (21.9)	1,107 (30.8)
High school/technical secondary school	130 (14.2)	898 (25.0)
College, bachelor's degree	48 (5.2)	444 (12.4)
Graduate degree	2 (0.2)	14 (0.4)
Health_status (%)		
Excellent	29 (3.2)	8 (0.2)
Good	256 (28.0)	242 (6.7)
Middle	537 (58.7)	1,586 (44.1)
Poor	89 (9.7)	1,298 (36.1)
Very poor	4 (0.4)	459 (12.8)
Chest_tightness_or_chest_pain = yes (%)	235 (25.7)	604 (16.8)
Difficulty_urinating = yes (%)	84 (9.2)	182 (5.1)
Memory_decline = yes (%)	566 (61.9)	2,181 (60.7)
Shortness_of_breath_with_activity_edema = yes (%)	183 (20.0)	501 (13.9)
Urinary_incontinence = yes (%)	27 (3.0)	60 (1.7)
Fractures_without_external_cause = yes (%)	11 (1.2)	82 (2.3)
Falls_without_external_force = yes (%)	98 (10.7)	329 (9.2)
Recurrent_joint_pain = yes (%)	237 (25.9)	866 (24.1)
Insomnia = yes (%)	384 (42.0)	1,342 (37.4)
Mobility_issues_slow lame = yes (%)	396 (43.3)	542 (15.1)
Dizziness	394 (43.1)	1237 (34.4)
Constipation = yes (%)	231 (25.2)	762 (21.2)
Source of the participants=community-based (%)	309 (33.8)	2,703 (75.22)
Living_environment = 2 (%)	835 (91.3)	3,389 (94.3)
Age [mean (SD)]	74.82 (7.67)	70.57 (6.45)
Self rated_health_score [mean (SD)]	4.98 (1.65)	6.91 (1.87)
Number_of_chronic_diseases [mean (SD)]	1.73 (1.40)	1.62 (1.30)
Number_of_medications_taken [mean (SD)]	2.40 (3.13)	2.25 (2.10)
Number_of_hospitalizations_in_the_past_year [mean (SD)]	0.80 (1.54)	0.55 (2.70)
Number_of_emergency_visits_in_the_past_year [mean (SD)]	0.75 (4.71)	0.51 (4.03)

Tips: the options for “Living environment” are as follows: 1 = large and medium-sized cities; 2 = suburban areas; 3 = townships and cities in the countryside; 4 = rural areas; 5 = other. The options for “source of the participants” are as follows: 0 = community-based, 1 = instruction-based and others.

### 3.3 Factors for “overall high IC”

The result of the univariate regression analysis and RF model regarding the propensity for a “Overall High IC” phenotype are summarized in Table 3 and Figure 2b, with corresponding LASSO regression results shown in Figures 2c, d. Fifteen variables reached statistical significance (*P* < 0.05) during this stage.

**Table 3:** Results of single-factor analysis and random forest model.

Variable	*B*	SE	Wald	DF	Exp(*B*)	EXP(*B*) 95%*CI*		*P*-value	MDA	MDG
						Down	Up			
Age	0.062	0.007	78.429	1	0.94	0.927	0.953	0	37.897899	146.048304
Main_occupation_in_life			15.355	3	–	–	–	0.002	14.982869	49.443343
Per_capita_disposable_monthly_income			44.98	6	–	–	–	0	26.148173	90.765559
Economic_status			15.213	2	–	–	–	0	18.236659	42.66528
Marital_status			6.652	5	–	–	–	0.248	21.469864	43.409711
Family_type			41.714	5	–	–	–	0	22.013216	70.193685
Educational_level			39.412	5	–	–	–	0	27.786356	88.165965
Health_status			71.172	4	–	–	–	0	44.700773	100.498161
Self-rated_health_score	0.233	0.038	36.636	1	1.262	1.17	1.361	0	67.23503	161.849186
Number_of_chronic_diseases	0.137	0.043	10.19	1	1.146	1.054	1.247	0.001	14.330473	62.393888
Number_of_medications_taken	0.006	0.021	0.089	1	1.006	0.966	1.049	0.766	14.935811	71.589632
Number_of_hospitalizations_in_the_past_year	−0.012	0.014	0.819	1	0.988	0.962	1.014	0.366	19.368333	48.152842
Number_of_emergency_visits_in_the_past_year	−0.004	0.01	0.145	1	0.996	0.977	1.016	0.704	10.261003	33.893509
Chest_tightness_or_chest_pain (yes)	0.249	0.119	4.399	1	1.283	1.016	1.619	0.036	5.078841	20.730508
Difficulty_urinating (yes)	0.112	0.176	0.406	1	1.119	0.792	1.58	0.524	6.165394	11.289486
Memory_decline (yes)	0.039	0.094	0.169	1	1.039	0.865	1.249	0.681	3.425075	25.507473
Shortness_of_breath_with_activity_edema (yes)	−0.05	0.127	0.152	1	0.952	0.742	1.221	0.696	5.67105	19.151656
Urinary_incontinence (yes)	0.118	0.296	0.159	1	1.125	0.63	2.01	0.69	2.304769	5.178271
Fractures_without_external_cause (yes)	−0.957	0.391	5.983	1	0.384	0.178	0.827	0.014	5.255279	4.067646
Falls_without_external_force (yes)	−0.286	0.157	3.33	1	0.751	0.552	1.021	0.068	2.16913	13.258086
Recurrent_joint_pain (yes)	−0.087	0.107	0.66	1	0.916	0.742	1.131	0.416	5.649872	21.452911
Insomnia (yes)	0.243	0.094	6.666	1	1.275	1.06	1.533	0.01	10.233569	26.439219
Mobility_issues_(slow/lame) (yes)	0.932	0.103	81.976	1	2.541	2.076	3.109	0	42.341554	63.712517
Dizziness (yes)	0.284	0.093	9.268	1	1.329	1.106	1.595	0.002	6.727233	25.497281
Constipation (yes)	0.085	0.108	0.623	1	1.089	0.881	1.346	0.43	2.084732	21.865274
Living_environment (yes)	−0.142	0.169	0.703	1	0.868	0.623	1.209	0.402	6.373461	11.728202
Intercept	4.1	1.524	7.244	1	60.37	–	–	0.007	–	–

**Figure 3:**
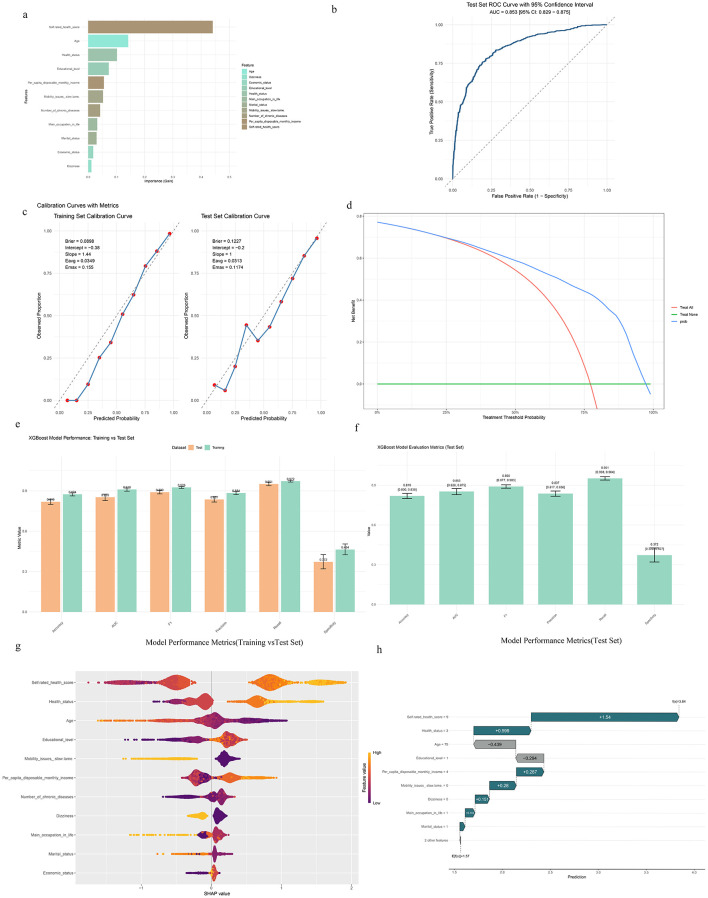
XGBoost model performance and visualization. **(a)** Horizontal bar chart of feature importance ranked by gain. **(b)** Receiver operating characteristic (ROC) curve with area under the curve (AUC). **(c)** Calibration curves for the training and test sets, with accompanying calibration metrics. **(d)** Decision curve analysis (DCA) of the model. **(e)** Grouped bar chart of model performance metrics for the training and test sets. **(f)** Bar chart of XGBoost model evaluation metrics on the test set. **(g)** Violin plots of SHAP values for different features showing feature distributions. **(h)** Bar chart of individual (NO.24) SHAP feature contributions to model predictions.

Specifically, univariate regression analysis identified several factors associated with “Overall High IC”: higher SRH (*OR* = 1.26, 95% *CI* = 1.17–1.36), the presence of multiple chronic diseases (*OR* = 1.146, 95% *CI* = 1.054–1.247), chest tightness and pain (*OR* = 1.283, 95% *CI* = 1.016–1.619), and mobility issues (*OR* = 2.541, 95% *CI* = 2.076–3.109). Notably, univariate regression analysis also showed that age was inversely associated with this profile (*OR* = 0.87, 95% *CI* = 0.80–0.95), indicating that the likelihood of belonging to this profile decreases with increasing age. The RF model achieved a minimum out-of-bag (OOB) error rate of 21.23% (ntree = 610, mtry = 3) after hyperparameter tuning and identified the five most important variables as self-rated health score (SRH), health status, mobility disability (slow/lame), age, and educational level (Figure 2b). Furthermore, LASSO regression model was applied to the training set to identify independent features. By integrating the results of RF model and LASSO regression model, 11 optimal features were selected: SRH, age, health status, educational level, monthly per capita disposable income, mobility issues (slow/lame), number of chronic diseases, primary lifetime occupation, marital status, economic status, and dizziness (Figure 3a).

### 3.4 Performance of the IC classification model and internal validation

#### 3.4.1 XGBoost model performance

The performance of the IC classification model was evaluated across four domains, including discrimination, clinical utility, calibration, and generalization. From the confusion matrix, as shown in Table 4, Figures 3b–d and Supplementary Table 4, the XGBoost model demonstrated robust performance in the training set, with both the AUC and accuracy exceeding 0.85 [*AUC* = 0.9104, 95% *CI* (0.8979–0.921); accuracy = 0.8742, 95% *CI* (0.8624–0.8849)]. Although the model exhibited lower specificity [0.4637, 95% *CI* (0.4250–0.5050)], the recall and *F*1-score were notably high at 0.9718, 95% *CI* (0.9651–0.9782) and 0.9258, 95% *CI* (0.9185–0.9325), respectively. In the independent test set, the model maintained robust performance with AUC of 0.8525, 95% *CI* (0.8284–0.8751) and accuracy of 0.8189, 95% *CI* (0.7997–0.8381). While the specificity and *F*1-score were slightly lower than those in the training set, the recall remained high at 0.3722, 95% *CI* (0.3204–0.4268) and 0.8902, 95% *CI* (0.8775–0.9030). Table 4 presents detailed goodness-of-fit tests for the XGBoost model, as well as calibration parameters derived from calibration curves. These results indicate that the model possesses strong discriminative capacity across both datasets. Furthermore, DCA of the test set revealed a clinical net benefit within a threshold probability range of 4%−97%, highlighting the high clinical utility (Figures 3e, f). Consider a medium-sized community with a resident population of 800 older adults. Assuming that the actual prevalence of the “Overall High IC” profile in this community is approximately 15% (i.e., 120 older adults genuinely exhibit this favorable profile). When the community health center uses this model for preliminary classification, it correctly identifies approximately 107 of the 120 truly high-IC individuals (sensitivity: 89.02%), only about 13 high-IC individuals being missed. However, among the 680 older adults who do not belong to this favorable profile, the model only correctly rules out approximately 253 individuals (specificity: 37.22%), meaning that about 427 individuals would be misclassified as “Overall High IC” and would require further objective assessment to confirm their actual IC status. Thus, this classification results should serve only as a reference for further comprehensive assessment.

**Table 4:** The corrected curve parameters of the training set and the test set.

Dataset	Training	Test
Dxy	0.820893976	0.705002542
C_ROC	0.910446988	0.852501271
R2	0.525850189	0.400821918
D	0.39745441	0.305746878
D_Chi_sq	1,254.968665	414.675526
D_p	0	0
U	0.020327353	0.003614706
U_Chi_sq	66.13279935	6.890696809
U_p	4.32987E−15	0.031893648
Q	0.377127057	0.302132172
Brier	0.089843363	0.122684659
Intercept	−0.383378379	−0.203642967
Slope	1.442085615	0.997230235
Emax	0.187295298	0.068661342
E90	0.079471792	0.05646704
Eavg	0.032933777	0.022826665
S_z	−5.692639559	1.704443592
S_p	1.2509E−08	0.088298246

From the perspective of resource optimization, a traditional “universal screening” strategy would require all 800 older adults to undergo time-consuming comprehensive assessments. In contrast, after applying our model for primary screening, approximately 534 individuals would be flagged as high-risk and proceed to the next stage of assessment, while approximately 266 low-risk individuals (representing 33% of the community) could be exempted from further screening.

These findings clearly delineate the clinical utility of our model: with its high sensitivity, it excels at capturing nearly 90% of high-risk individuals, ensuring that the majority of those needing attention are not missed, making it highly suitable as a primary risk-screening tool in community settings. Conversely, the low specificity implies that older adults flagged as positive still require confirmation through subsequent objective functional assessments; thus, the model should not be used as a standalone diagnostic tool.

#### 3.4.2 Visualization of results

Risk factors were ranked in descending order based on the mean absolute SHAP values calculated across the test set. As shown in Figure 3g, the 11 most influential features were: self-rated health score, age, health status, educational level, average monthly income, mobility issues (slow/lame), number of chronic diseases, primary lifetime occupation, marital status, economic status, and the presence of dizziness. The descriptions and coding directions of the 11 most influential features shown in Figure 3g are provided in Supplementary Table 5.

To demonstrate the clinical interpretability at the individual level, a SHAP force plot was generated for Subject No. 24 (Figure 3h). For this individual, the model's baseline expected value [*E*–*f* (x)] was 1.57, while the final output [*f* (*x*)] reached 3.84. This significantly higher value indicates a strong propensity for the “Overall High IC”. A SRH of 9 was the primary driver increasing the model performance. Other contributing factors included poor health status, a monthly disposable income of 3,000–5,999 RMB, the absence of mobility issues or dizziness, a primary occupation involving light physical labor, and marital status. In contrast, age (79 years) was the feature contributing most negatively to the model, while education level reduced the score by an additional 0.294 units.

#### 3.4.3 Interactive platform construction

**Figure 4:**
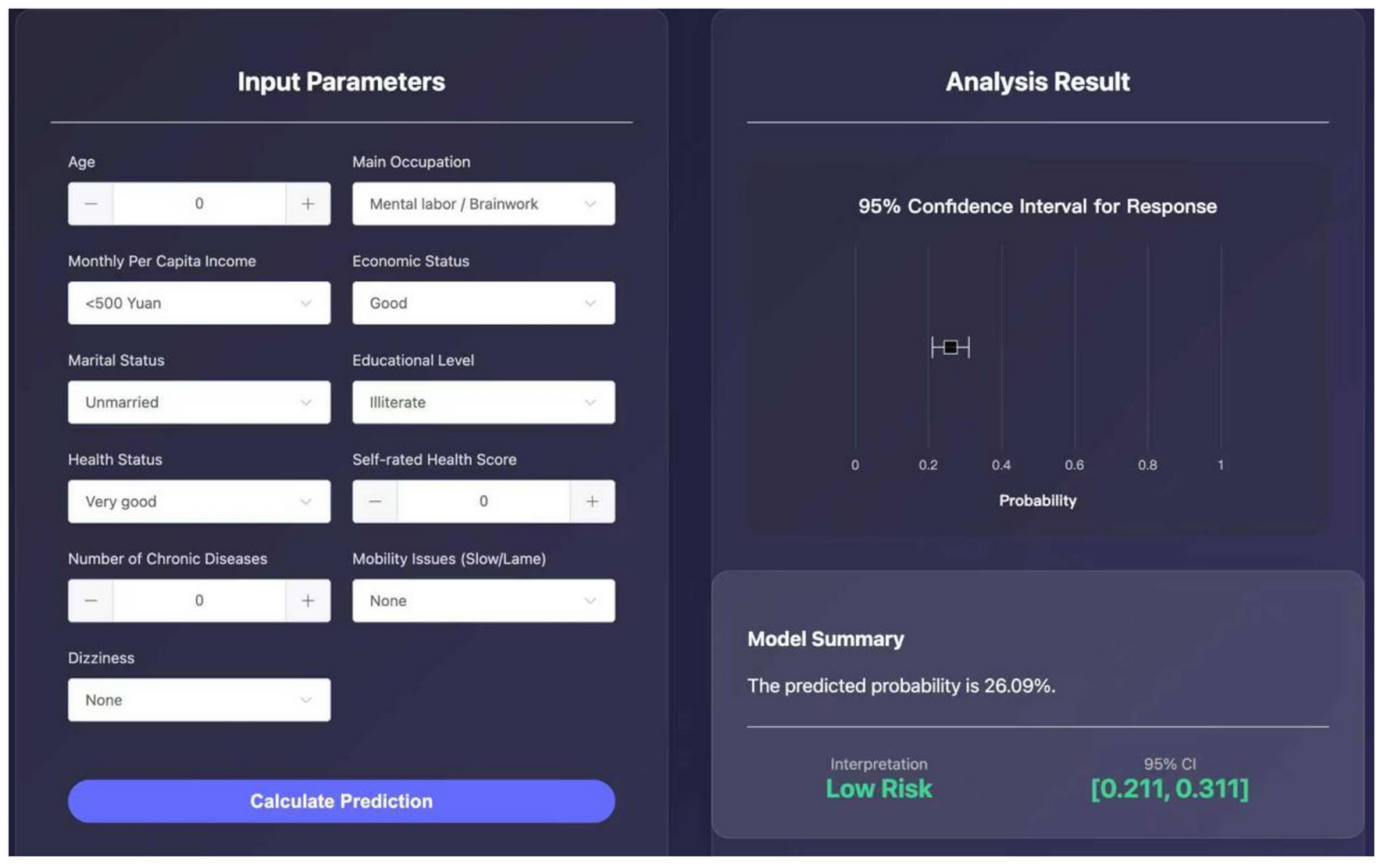
The deployed web-based platform for IC profile classification. Two-panel interface displaying input parameters on the left, including age, occupation, income, economic and marital status, education, health, chronic diseases, mobility, and dizziness; and a prediction analysis on the right showing a probability chart with a 95% confidence interval, summary indicating a predicted probability of 26.09%, classified as low risk with a confidence interval between 0.211 and 0.311.

The optimized XGBoost model was deployed as a web-based, interactive clinical decision support tool. After entering individual features, the system generates the obtained IC class and its associated probability within 3 s. To enhance transparency, the platform also provides a visualization window that delineates the specific contribution of each feature (Figure 4). Moreover, data security protocols were implemented to ensure user privacy protection. Specifically, the tool does not collect any personally identifiable information. Individual records are identified solely by an auto-generated study ID, and no names, national identifiers, contact details, or other personal data are entered into or stored by the platform.

## 4 Discussion

This study identified two latent IC profiles among 4,508 Chinese older adults recruited between December 2023 and December 2024, labeled as “Overall Low IC” and “Overall High IC”. Notably, these identified profiles primarily reflect an overall gradient of IC, with one profile consistently showing higher scores across all domains and the other consistently showing lower scores, rather than distinct domain-specific patterns. This classification represents a data-driven result from LPA rather than an artifact of arbitrary total-score stratification based on predefined cutoffs (e.g., a mean or median split). These findings help fill the gap in research on LPA-based IC profiles among large Chinese older populations and provide more contemporary IC data. Furthermore, an XGBoost-based analytical model was developed using cross-sectional data, including self-rated health, age, health status, educational level, and monthly per capita disposable income. This analytical model achieved an AUC of 0.8525 (95% *CI*: 0.8284–0.8751) and was implemented in an interactive online platform, providing a proof-of-concept for a simplified and digitally approach to IC profile assessment.

An interesting finding from previous studies is that various analytical methods (e.g., LCA and cluster analysis) have consistently revealed a pattern of heterogeneity in IC among older adults (Du et al., 2026; Wang et al., 2026; Cheng et al., 2026; Chen et al., 2025; Qiao et al., 2025; Kim et al., 2024). For example, Du et al. (2026) applied LCA to 307 older adults from two hospital in Shanxi Province, China, identifying three profiles “high motor-medium psychological,” “low motor-low sensory-high psychological,” and “relatively low.” Although the number and labels of latent classes vary across studies, a broadly consistent distinction between higher and lower IC profiles can be observed, which aligns with the pattern reported in our study. Building on this, we applied the LPA method to a larger and more recent dataset of older adults across 24 provinces in China to validate this trend. Our findings provide new evidence for the classification of heterogeneous IC levels among older adults and offer potential support for the development of future classification standards.

Notably, this study also explored the associations between various factors and group classifications. Among these factors, self-rated health (SRH) showed the strongest association, with lower SRH scores being more likely to be observed in the “Overall Low IC” group. This finding is consistent with previous studies demonstrating a close relationship between IC and SRH. Specifically, Shiratsuchi et al. (2025) reported that older adults with both frailty and low IC had 3.55 times higher odds of reporting poor SRH compared with those who were robust and had high IC (*OR* = 3.55, 95% *CI*: 1.34–9.36). Furthermore, based on the Longitudinal Study on Aging data from Taiwan Province, it was also discovered that age-specific patterns of impaired intellectual capabilities were associated with poor self-assessed health (Chu et al., 2025). These findings suggest that SRH and IC are closely interconnected. Therefore, SRH may serve as a valuable complementary measure when evaluating IC in older adults, helping to provide a more comprehensive understanding of their overall health status. Moreover, age and education level were also associated with IC classification (Cui et al., 2026). Specifically, higher age was linked to “Overall Low IC”, consistent with the WHO healthy aging framework (Adamakidou et al., 2026; Yang et al., 2026; Gou et al., 2026), which describes age-related declines across multiple domains of IC. Additionally, our study also indicates that a higher education level and a higher salary are both associated with “Overall High IC”, which is consistent with the findings of Beyene et al. (2026). As emphasized by the WHO (2020), IC is not only dependent on current physiological status but is also influenced by experiences. Individuals with higher educational attainment generally demonstrate higher levels of health literacy and awareness of both physical and mental health, which may facilitate the adoption of health-promoting behaviors and proactive health management, thereby contributing to the maintenance and enhancement of IC over time (Mihaiescu-Ion et al., 2026; Su et al., 2026). Therefore, providing targeted health education for older adults with lower educational attainment may contribute to the promotion of healthy aging and the maintenance of IC.

To identify the stratified classes, the study developed an interactive online IC assessment tool based on XGBoost analytical model, which enabled rapid classification of IC status. Compared with a traditional questionnaire, the test time was reduced from 30 min to 5 min, which not only significantly improved efficiency in practical applications but also facilitated the collection of relevant data from older adults. This platform is developed as a proof-of-concept for classifying IC status in older adults, demonstrating the feasibility of rapid IC assessment in routine clinical settings, intended for use by community healthcare workers and general practitioners.

However, this study also has several limitations. First, the cross-sectional design and reliance on self-reported data may introduce recall bias and constrain the generalizability of classification model's findings to populations with diverse cultural backgrounds, different care settings, and other older adult cohorts. Second, the online classification platform was constructed solely using the XGBoost algorithm and the stability and reproducibility of the latent profiles were examined only within the current sample, and that the model without external independent evidence of generalizability. The model captures covariation signals between self-reports and objective measurements rather than novel, independent etiological information. Meanwhile, the psychological domain, being part of the outcome, may share method variance with subjective predictors sensitive to emotional states, and we recommend future studies control for current depressive symptoms when using such predictors. This study also may be subject to selection bias. Specifically, the 30–40-min face-to-face survey may have excluded individuals unable to complete the survey, those with communication barriers, and those who were bedridden. Therefore, the IC profiles identified should be interpreted with reference to the accessible, community-based population rather than the full older adults. The random split strategy adopted in this study precluded further stratified or subgroup analyses, which may limit the assessment of model performance across specific populations. Future research should employ public datasets to refine the classification algorithm, and recruit more diverse populations outside China to further assess the generalizability of the findings. At the same time, future studies should consider validation using longitudinal data and independent external cohorts before wider implementation.

## 5 Conclusion

This study identified two IC profiles “Overall High IC” and “Overall Low IC” among 4,508 Chinese older adults and developed an XGBoost-based classification model with an accompanying online platform to enable rapid profiles classification. These findings highlight the heterogeneity of IC in older populations and suggest a simplified digital approach to IC profile assessment. Future studies with external validation in prospective cohorts are warranted to further evaluate the generalizability and value of this approach.

## Data Availability

The raw data supporting the conclusions of this article will be made available by the authors upon reasonable request.
