# Supplementary material for: Latent profile analysis and analytical model construction based on heterogeneity of intrinsic capacity in Chinese older adults
Source: Front Aging Neurosci. 2026 Jul 28;18:1858286. doi: 10.3389/fnagi.2026.1858286 (PMC13457452; doi:10.3389/fnagi.2026.1858286)
Supplement: Supplementary file 1 [file Data_Sheet_1.docx]

**Supplementary Material 1:** **The survey instrument used in this study**

**1.1 Basic demographic characteristics**

The basic demographic characteristics were collected through the questionnaires developed by the research team itself. These questionnaires included: general demographic information (such as age, gender, monthly income, educational level, etc.)

**1.2 The Short Physical Performance Battery (SPPB)**

The Short Physical Performance Battery (SPPB) consists of (i) balance, (ii) walking speed, and (iii) five chair inversions. The balance test is divided into three parts. First, patients are required to stand with their feet together for 10 seconds. If they succeed, they receive one point and proceed to the next step; if not, they receive 0 points. In the second step, patients are required to stand with one foot's heel touching the side of the other big toe for 10 seconds. If they succeed, they receive one point and proceed to the next step. In the third step, patients are instructed to align their heels with their toes and stand for 10 seconds. If they stand for 10 seconds, they receive 2 points; if their time is between 3 and 9.99 seconds, they receive 1 point; if less than 3 seconds, they receive 0 points.

In the walking speed test, patients are required to walk 4 meters twice at a normal speed and record the time taken. The lower value of the walking time is the final score. If patients walk for less than 4.82 seconds, they receive 4 points; if between 4.82 and 6.2 seconds, they receive 3 points; between 6.21 and 8.70 seconds, they receive 2 points, and more than 8.7 seconds, they receive 1 point; if they fail to complete the 4-meter walk, they receive 0 points.

In the chair standing test, patients cross their arms over their chest and stand up from the chair once. If they cannot stand, the test is terminated and they receive 0 points. Otherwise, patients are instructed to stand up from the chair as quickly as possible five times without using their arms. If the time is ≤ 11.19 seconds, they receive 4 points. Between 11.2 and 13.69 seconds, they receive 3 points. Between 13.7 and 16.69 seconds, they receive 2 points. If the time is > 16.7 seconds, they receive 1 point; if more than 60 seconds, they receive 0 points. All patients underwent the SPPB test at baseline and their scores were calculated. Each patient took approximately 15–20 minutes to complete the SPPB. If the SPPB score is < 10, the diagnosis is weakness.

**1.3 the Geriatric Depression Scale Short Form (GDS-15)**

This study used the Geriatric Depression Scale Short Form (GDS-15) to assess the mental health status of the elderly. This scale was developed by Sheikh and Yessavici and later translated into Chinese and verified by Mei. The characteristics of GDS-15 are as follows: (1) This scale is specifically designed for the elderly and is currently one of the most commonly used tools for screening depression in the elderly; (2) It contains 15 dichotomous questions that can be answered with "yes" or "no", which is easier to answer and more suitable for the elderly to respond to. The total score ranges from 0 to 15, with a higher score indicating a higher degree of depression. GDS-15 has high sensitivity and specificity in clinical depression detection, and has high reliability among Chinese elderly people. A GDS score between 0 and 4 indicates no depression; a score of 5 or above indicates possible depression. The higher the GDS score, the more severe the depression is, and the worse the mental health status is. The psychological domain was assessed using the GDS-15, where higher raw scores indicate more severe depressive symptoms and poorer psychological capacity. To ensure consistency in score direction across all IC domains, scores were reverse-coded as follows:

$$Adjusted psychological score = 15 - raw GDS-15 score$$

Higher adjusted scores indicate lower depressive symptomatology and better psychological capacity.

**1.4** **The Minimum Mental State Examination (MMSE)**

MMSE is a brief and structured screening test for global cognition. This test consists of 30 items, divided into 6 categories: time and spatial orientation; memory (reciting three words), attention and calculation (continuous subtraction or reverse spelling, recalling previously memorized words); language (identification of two objects, repetition of short sentences; sentence comprehension; sentence writing) and practical construction (designing a text). The score is given by the number of correctly completed items (0 to 30). The lower the score, the worse the performance and the more severe the cognitive impairment.

**1.5 Sensory function**

Sensory function included both vision and hearing assessments: (1) Distance vision was tested using a simplified E chart protocol. Scores ranged from 0 to 3 based on the participant’s ability to identify a small or large E at varying distances (each eye scored separately). 3 points were given if the participant could answer 3 out of 4 small Es’ directions correctly at 3 meters; 2 points were given for identifying ¾ large Es correctly at 3 meters; and 1 point was given for identifying ¾ large Es correctly at 1.5 meters; Near vision was scored (0 ~ 3) using a near vision chart (3 rows with decremental font sizes). (2) Hearing was assessed using whisper test. The assessor stood at arm's length on one side of the subject, asked the subject to press the opposite tragus, and whispers four common and unrelated words. The subject is asked to repeat the above words. If the subject can repeat ≥ 3 words, the hearing on this side is normal; different words are used to assess the opposite side. 2 points were assigned to each ear (possible scores were 0, 2, 4 points for hearing). This protocol yielded a 0–10 points scale for sensory function.
