# Supplementary material for: Latent profile analysis and analytical model construction based on heterogeneity of intrinsic capacity in Chinese older adults
Source: Front Aging Neurosci. 2026 Jul 28;18:1858286. doi: 10.3389/fnagi.2026.1858286 (PMC13457452; doi:10.3389/fnagi.2026.1858286)
Supplement: Supplementary file 3 [file Table_1.docx]

**Supplementary Material**

**Table 1** LPA analysis after data standardization

| Class | AIC | BIC | aBIC | Entropy | LMIR | BLRT | Class Probability |
| --- | --- | --- | --- | --- | --- | --- | --- |
| 1 | 85506.033 | 85573.07 | 85541.293 | - |  |  |  |
| 2 | 80699.188 | 80806.447 | 80755.603 | 0.846 | 0 | 0 | 0.188/0.812 |
| 3 | 78905.157 | 79052.638 | 78982.728 | 0.899 | 0.0613 | 0 |  |
| 4 | 77510.89 | 77698.593 | 77609.617 | 0.874 | 0 | 0 |  |
