# Supplementary material for: Latent profile analysis and analytical model construction based on heterogeneity of intrinsic capacity in Chinese older adults
Source: Front Aging Neurosci. 2026 Jul 28;18:1858286. doi: 10.3389/fnagi.2026.1858286 (PMC13457452; doi:10.3389/fnagi.2026.1858286)
Supplement: Supplementary file 4 [file Table_2.docx]

**Supplementary Table**

**Supplementary Table 2. Sensitivity Analysis: Agreement Between LPA Classification and Total-Score-Based Binary Classification**

| **Panel A. Classification Agreement Between LPA and Total-Score Splits** | | | |
| --- | --- | --- | --- |
|  | **Mean Split (cut = 63.27)** | | **Median Split (cut = 66.0)** |
| Overall concordance, % | 76.37% | | 68.53% |
| Cohen's κ | 0.47 | | 0.37 |
|  | | | |
| **Confusion matrix** |  | |  |
| Both Low | 1,093 | | 1,093 |
| Split-Low / LPA-High (false low) | 1,424 | | 1,896 |
| Split-High / LPA-Low (false high) | 0 | | 0 |
| Both High | 3,508 | | 3,036 |
|  | | | |
| **Panel B. Domain-Level Means by Agreement Status (Mean Split vs. LPA)** | | | |
| **Domain** | **Agree-Low (n = 1,093)** | **Disagree (n = 1,424)** | **Agree-High (n = 3,508)** |
| Cognitive | 10.92 | 12.20 | 13.06 |
| Psychological | 4.09 | 6.54 | 9.80 |
| Sensory | 18.59 | 22.50 | 27.12 |
| Vitality | 8.23 | 11.63 | 13.11 |
| Locomotor | 4.18 | 6.17 | 7.27 |
| **Total score** | **46.02** | **59.05** | **70.36** |
|  | | | |
| **Panel C. Disagree Group Domain Means as Percentage of Agree-High Group** | | | |
| **Domain** | **Disagree / Agree-High (%)** | | |
| Cognitive | 93.4% | | |
| Vitality | 88.8% | | |
| Locomotor | 84.8% | | |
| Sensory | 83.0% | | |
| Psychological | 66.8% | | |

Note. LPA = latent profile analysis. The total IC score was computed as the sum of five domain scores. Mean split cutoff = 63.27; median split cutoff = 66.0. LPA Class 1 corresponds to "Overall Low IC" and LPA Class 2 to "Overall High IC." The "Disagree" group includes participants whose total score fell below the mean cutoff but who were assigned to Class 2 (Overall High IC) by LPA. Panel C ranks the five domains by the degree to which the Disagree group's domain means approximate those of the Agree-High group, revealing that cognitive function was the most preserved domain among these individuals.
