# Supplementary material for: Latent profile analysis and analytical model construction based on heterogeneity of intrinsic capacity in Chinese older adults
Source: Front Aging Neurosci. 2026 Jul 28;18:1858286. doi: 10.3389/fnagi.2026.1858286 (PMC13457452; doi:10.3389/fnagi.2026.1858286)
Supplement: Supplementary file 5 [file Table_3.docx]

**Supplementary Table**

**Supplementary Table 3. Average Posterior Probabilities and Classification Quality for the 2-Class LPA Solution**

| **Latent Class** | **n** | **%** | **Average posterior probability** | | **AvePP** |
| --- | --- | --- | --- | --- | --- |
|  |  |  | **Class 1** | **Class 2** |  |
| Class 1 | 1,093 | 18.1 | **0.895** | 0.105 | 0.895 |
| Class 2 | 4,932 | 81.9 | 0.031 | **0.969** | 0.969 |
|  | | | | | |
| **Classification Quality Indices** | | | | | |
| Entropy | | | 0.846 | | |
| Overall AvePP | | | 0.955 | | |
| % maxPosterior < 0.95 | | | 18.95% (n = 1,142) | | |
| % maxPosterior < 0.90 | | | 14.39% (n = 867) | | |
| % maxPosterior < 0.80 | | | 8.90% (n = 536) | | |
| % maxPosterior < 0.70 | | | 5.31% (n = 320) | | |
|  | | | | | |
| **Per-Class Classification Uncertainty** | | | | | |
|  | | | **Class 1** | **Class 2** | |
| n | | | 1,093 | 4,932 | |
| AvePP | | | 0.895 | 0.969 | |
| MaxPosterior < 0.80 | | | 22.4% | 5.9% | |
| MaxPosterior < 0.70 | | | 14.1% | 3.4% | |

Note. AvePP = average posterior probability for the assigned class. Diagonal values (bold) are the average posterior probabilities for each latent class. Classification uncertainty is the proportion of individuals whose maximum posterior probability falls below the specified threshold.
