# Supplementary material for: Latent profile analysis and analytical model construction based on heterogeneity of intrinsic capacity in Chinese older adults
Source: Front Aging Neurosci. 2026 Jul 28;18:1858286. doi: 10.3389/fnagi.2026.1858286 (PMC13457452; doi:10.3389/fnagi.2026.1858286)
Supplement: Supplementary file 6 [file Table_4.docx]

**Supplementary Table**

**Supplementary Table 4** XGBoost Model Evaluation Metrics

| Variable | Train | Test |
| --- | --- | --- |
| AUC(95%CI) | 0.9104 (0.8979-0.921) | 0.8525 (0.8284-0.8751) |
| Accuracy(95%CI) | 0.8742 (0.8624-0.8849) | 0.8189 (0.7997-0.8381) |
| Precision(95%CI) | 0.8840 (0.8717-0.8950) | 0.8366 (0.8166-0.8562) |
| Recall(95%CI) | 0.9718 (0.9651-0.9782) | 0.9511 (0.9385-0.9639) |
| Specificity(95%CI) | 0.4637 (0.4250-0.5050) | 0.3722 (0.3204-0.4268) |
| F1(95%CI) | 0.9258 (0.9185-0.9325) | 0.8902 (0.8775-0.9030) |
