# Supplementary material for: Latent profile analysis and analytical model construction based on heterogeneity of intrinsic capacity in Chinese older adults
Source: Front Aging Neurosci. 2026 Jul 28;18:1858286. doi: 10.3389/fnagi.2026.1858286 (PMC13457452; doi:10.3389/fnagi.2026.1858286)
Supplement: Supplementary file 7 [file Table_5.docx]

**Supplementary Table**

**Supplementary Table 5. Variable definitions, coding schemes, and descriptive statistics (N = 4508)**

| **No.** | **Variable** | **Label** | **Type** | **Coding / Categories** |
| --- | --- | --- | --- | --- |
| 1 | Self-rated health score | Self-rated health | Ordinal (0–10) | 0 = worst health; 10 = best health |
| 2 | Health status | Objective health status | Categorical (5 levels) | 1 = Very poor; 2 = Poor; 3 = Middle; 4 = Good; 5 = Excellent |
| 3 | Age | Age | Continuous (years) | Range: 60–100 |
| 4 | Educational level | Education | Categorical (6 levels) | 1 = Illiterate; 2 = Primary school; 3 = Junior high school;4 = High school / Technical secondary school;5 = College / Bachelor’s degree; 6 = Graduate degree |
| 5 | Mobility issues (slow / lame) | Mobility impairment | Binary | 0 = No; 1 = Yes |
| 6 | Per capita disposable monthly income | Monthly income | Categorical (7 levels) | 1 = < 500 Yuan; 2 = 500–999 Yuan; 3 = 1,000–1,999 Yuan;4 = 2,000–2,999 Yuan; 5 = 3,000–5,999 Yuan;6 = 6,000–9,999 Yuan; 7 = ≥ 10,000 Yuan |
| 7 | Number of chronic diseases | Chronic disease count | Continuous (count) | Range: 0–11 |
| 8 | Dizziness | Dizziness | Binary | 0 = No; 1 = Yes |
| 9 | Main occupation in life | Lifetime occupation | Categorical (4 levels) | 1 = Mental labor; 2 = Light physical labor;3 = Heavy physical labor; 4 = Other |
| 10 | Marital status | Marital status | Categorical (6 levels) | 1 = Unmarried; 2 = Married, living together;3 = Married, separated; 4 = Divorced;5 = Widowed; 6 = Others |
| 11 | Economic status | Self-rated economic status | Categorical (3 levels) | 1 = Good; 2 = middle; 3 = Poor |

Note. All variables were included as predictors in the XGBoost model for intrinsic capacity. SHAP (SHapley Additive exPlanations) values were computed using the "shapviz" R package.
