# Supplementary material for: Latent profile analysis and analytical model construction based on heterogeneity of intrinsic capacity in Chinese older adults
Source: Front Aging Neurosci. 2026 Jul 28;18:1858286. doi: 10.3389/fnagi.2026.1858286 (PMC13457452; doi:10.3389/fnagi.2026.1858286)
Supplement: Supplementary file 8 [file Supplementary_file_1.docx]

**Supplementary Material**

**Figure 1** LPA analysis after data standardization
